# Fine Morphology of Antennal and Ovipositor Sensory Structures of the Gall Chestnut Wasp, *Dryocosmus kuriphilus*

**DOI:** 10.3390/insects12030231

**Published:** 2021-03-09

**Authors:** Milos Sevarika, Marco Valerio Rossi Stacconi, Roberto Romani

**Affiliations:** 1 Department of Agricultural, Food and Environmental Sciences, University of Perugia, 06121 Perugia, Italy; msevarika@gmail.com; 2 Research and Innovation Center, Fondazione Edmund Mach, S. Michele all’Adige, 38098 Trento, Italy; marcovalerio.rossistacconi@fmach.it

**Keywords:** sensilla, gall-inducting insects, morphology, ultrastructure, SEM, TEM, Cynipidae, Hymenoptera

## Abstract

**Simple Summary:**

Hymenoptera encompass a large group of insects with different habits, ranging from phytophagy to parasitic/predatory lifestyles. This is also true in the superfamily Cynipoidea, where phytophagy becomes highly specialized towards the exploitation of specific plant tissues (i.e., buds), leading to the induction of galls. In this paper, we investigated the organization of antennal and ovipositor sensory structures in the chestnut gall wasp, *Dryocosmus kuriphilus*. This insect became a major threat to chestnut production in Italy in the last 15 years. We investigated only females (this is a parthenogenetic species with thelytoky), and on the antennae we found several sensilla with the clear functional specialization to different groups of stimuli, with almost no overlapping among each sensilla. Similarly, specialization was also found on the ovipositor where groups of gustatory and mechanoreceptive sensilla were observed. This information represents an advancement in the knowledge of this pest, which may be useful to understand the biological role of plant derived chemical cues or to implement new control methods.

**Abstract:**

*Dryocosmus kuriphilus* is a gall-inducing insect, which can cause significant damage on plants of the genus *Castanea* Mill., 1754. Antennae and ovipositor are the main sensory organs involved in the location of suitable oviposition sites. Antennal sensilla are involved in the host plant location, while ovipositor sensilla assess the suitability of the ovipositional bud. On both organs, diverse sensillar organs are present. Here, the distribution and ultrastructural organization of the sensilla were investigated by scanning and transmission electron microscopy. The antennae of *D. kuriphilus* are filiform and composed of 14 antennomeres, with the distal flagellomere bearing the highest number of sensilla. On the antennae, 6 sensilla types were found; sensilla chaetica, campaniformia, coeloconica-I, coeloconica-II, trichoidea and placoidea. The sensilla placoidea and trichoidea were the most abundant types. On the external walls of the ovipositor, gustatory and mechanoreceptive sensilla were observed. Internally, the egg channel hosted two additional sensory structures. The putative functional role of each sensilla in the context of insect’s ecology is discussed as well as the ovipositional mechanism used by this insect.

## 1. Introduction

Gall-inducing insects are considered the most highly specialized herbivores, as they are able to cause development of specialized plant tissue. [1,2,3,4]. Galls provide the inducer and its progeny with food, protection from abiotic stress and natural enemies [5].

As a result of their high specialization, they have a narrow host range, attacking specific organs in one or a few related species [1,6]. However, several species display a host range across different plant families [1].

The total number of extant gall-inducing insect species is estimated at approximately 211,000 [6]. They are found within seven orders, with the highest number recorded among Diptera and Hymenoptera [7]. 

As representative of these specific phytophagous insects, the order Hymenoptera includes the family Cynipidae, the so-called gall wasps. Most of the cynipid gall-inducers do not have economic importance [4]. A remarkable exception is *Dryocosmus kuriphilus* Yasumatsu, the chestnut gall wasp (CGW), an oligophagous species that develops on plants of the genus *Castanea*. Native to China, *D. kuriphilus* has spread over Korea [8], USA [9] and lately Europe [10,11,12,13,14]. European population of *D. kuriphilus* results from a single introduction event [15].

*Dryocosmus kuriphilus* is able to reduce fruit yield by up to 80% [16]. This is mainly done through inhibition of fruit development and flowering, which in some cases can contribute to tree mortality [4]. For this species, the literature reports a wealth of studies dealing with taxonomy, biology, ecology as well as its biological control using introduced parasitoids belonging to the genus *Torymus* Dalman, 1820 (Hymenoptera: Torymidae). In comparison, little is known about morpho-functional adaptation of the *D. kuriphilus* in relation to such a specialized phytophagous habitus.

It is generally known that communication among insects is mediated with several cues, with chemicals playing a prominent role [17]. The importance of these cues on insect behaviour is well documented among several Hymenopteran species [18,19].

*Dryocosmus kuriphilus* displays peculiarities regarding its chemical ecology, namely it does not show attractiveness towards the undamaged host seedling, intact or freshly mechanically damaged twigs. On the contrary, it expresses attraction to the twigs with old mechanical damage [20].

Chemical cues are perceived with various sensilla located primarily on the insect antennae, which are the location of most of the olfactory sensilla. Chemosensory sensilla are also located on the insect ovipositor, whose function is associated with the selection of the oviposition site. Cynipids lay eggs in the apical portion of the bud, without reaching the subepidermal cells [21,22]. Once hatched, the larva induces the differentiation of the larval chamber. Simultaneously with the larval chamber differentiation, various chemical changes inside host plant occurs which affect plant tannins and phenolics profile [23].

To date, only a few studies investigating the fine structure of the antennal and ovipositor sensilla in Cynipoidea wasps are available in the literature [24,25]. Moreover, such studies have been conducted using scanning electron microscopy (SEM), thus lacking information on the sensillar ultrastructural organization. In a preliminary investigation carried out on *D. kuriphilus* female antennae and ovipositor, SEM data for some sensilla types were described (sensilla placoidea, sensilla trichoidea, sensilla chaetica and one type of ovipositor sensilla) [26]. Here, we report the occurrence of several new sensilla types present on the antenna and ovipositor. Moreover, ultrastructural investigation using transmission electron microscopy was carried out. Additionally, we provide a detailed map of the location and arrangement of the sensilla and discuss their potential role with respect to their morphological features. Lastly, we hypothesize the potential oviposition mechanism.

## 2. Materials and Methods

### 2.1. Insects

Adult female individuals of *D. kuriphilus* were obtained from dry galls randomly collected in various chestnut forests located in Umbria region of Italy, which were severely damaged by the CGW during March 2017 and March 2018. Galls were kept in netted cages (Kweekkooi 30 × 30 × 30 cm, Vermandel) placed in a climatic chamber (24 ± 1 °C; 12 h:12 h L:D) and checked daily for adult emergence.

### 2.2. Scanning Electron Microscopy (SEM)

Scanning electron microscopy observations were carried out on 10 adult female individuals. Insects were anaesthetized through low-temperature exposure (−18 °C for 3 min) and placed in 50% alcohol. To obtain a complete view of all the antennal structure, antennae (*n* = 10) were removed from the head capsule. To properly orientate antennae, intact heads (*n* = 5) were mounted in their natural position. Obtained specimens were processed in a series of graded ethanol, from 50% to 99%, with 10 min for each dehydration step. After dehydration, absolute (99%) ethanol was substituted with pure HMDS (Hexamethyldisilazane, Sigma-Aldrich, Dorset, UK) and the specimens were allowed to dry under a hood, at room conditions; this step was repeated twice. Samples were mounted on aluminium stubs, taking care to place them with different orientations to obtain a clear view of the ventral, dorsal and lateral sides of the antennomeres. Mounted specimens were gold-sputtered using a “Balzers Union^®^ SCD 040” unit (Balzers, Vaduz, Liechtenstein). The observations were carried out using a Philips^®^ XL 30 (Thermo Fischer Scientific, Hillsboro, OR, USA) operating at 7–10 kV, WD 9–10 mm.

### 2.3. Transmission Electron Microscopy (TEM)

For transmission electron microscopy (TEM) observations, 10 female individuals were anaesthetized by exposure to cold temperatures (−18 °C) for 100 s, then immediately transferred in a glutaraldehyde/paraformaldehyde solution (2.5% in 0.1 M cacodylate buffer +5% sucrose, pH 7.2–7.3). As the antennae in Cynipidae comprised more than 12 antennomeres, to improve fixative penetrations the antennae were separated on singular antennomere and resulting specimens were left at 4 °C for 24 h. Then, the specimens were washed twice in 0.1 M cacodylate buffer +5% sucrose, pH 7.2–7.3 for ten minutes. Afterwards, the specimens were postfixed in 1% OsO4 (osmium tetroxide) for 1 h at 4 °C and rinsed in the same buffer. Dehydration in a graded ethanol series from 60% to 99%, was followed by embedding in Epon-Araldite with propylene oxide as bridging solvent. Thin sections were taken with a diamond knife on a 2188 Ultratome Nova ultramicrotome (LKB^®^, Stockholm, Sweden), and mounted on formvar coated 50 mesh grids. Then, sections on grids were stained with uranyl acetate (20 min, room temperature) and lead citrate (5 min, room temperature). Finally, the sections were investigated with a Philips^®^ EM 208 (Thermo Fischer Scientific). Digital pictures (1376 × 1032 pixels, 8b, uncompressed greyscale TIFF files) were obtained using a high-resolution digital camera MegaViewIII (SIS^®^) connected to the TEM.

### 2.4. Antennal Mapping and Measurements

Data were obtained from antennae prepared as described above for the SEM protocol and observed using a Philips^®^ XL 30 and JCM-6000 Neoscope™ (Nikonmetrology NV, Europe, Leuven, Belgium). Twenty high magnification pictures of the different antennal regions were taken and mounted together in a single image to obtain a single, high-resolution picture showing details of a given antennal side. Each antennal side was analyzed with regards to the type of sensilla present, their abundance and their morphometrical features (i.e., length of the shaft, diameter of the shaft base), taking care not to consider artefacts and/or unmeasurable structures (i.e., tilted or broken sensilla). All measurements were made using ImageJ [27]. Mapping of the observed sensilla was done in Adobe Illustrator CC.

Within Hymenoptera, antennal sensilla occur in various shapes, therefore several classification methods were used to describe them. Here we referred to the nomenclature proposed in those papers where sensilla were investigated both for their external (SEM) and internal organization [28,29,30].

## 3. Results

**Figure 1:**
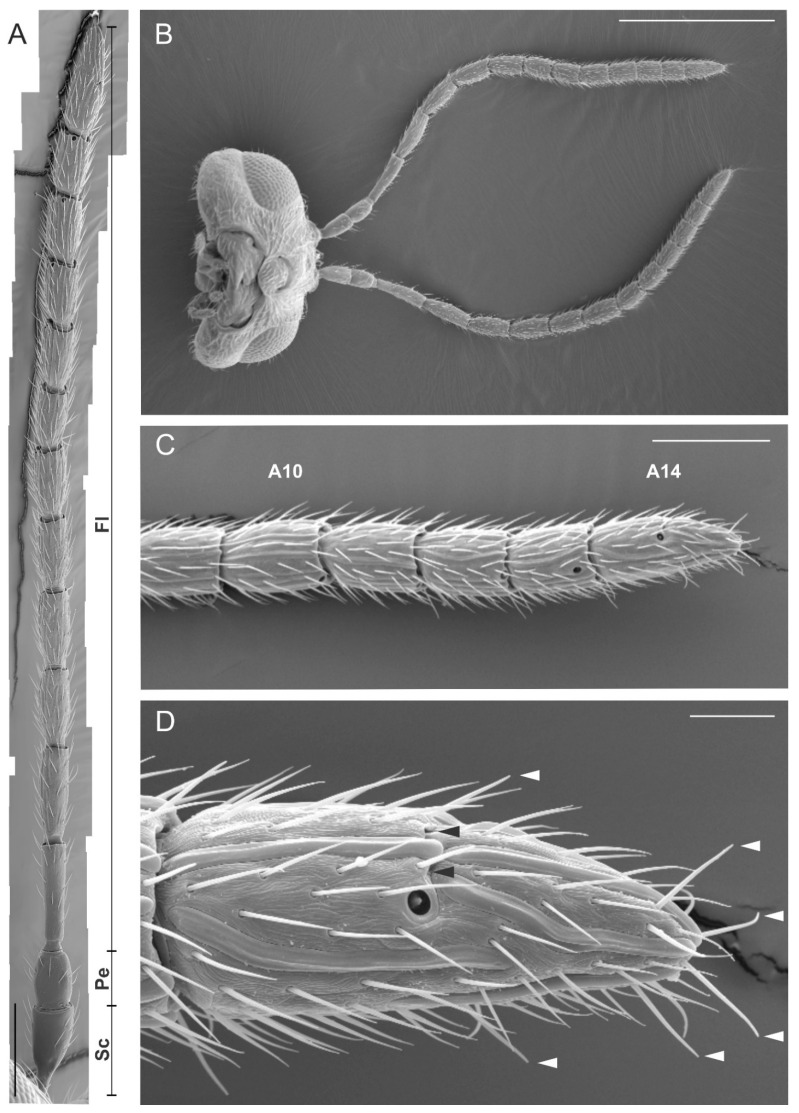
Scanning electron microscopy (SEM) micrographs showing the filiform antenna of *Dryocosmus kuriphilus* female. (**A**) General view of the antenna showing the scape (Sc), the pedicel (Pe) and the flagellum (Fl). The length (mean ± SD) of each antennomere was: Sc = 90.9 ± 5.1; Pe = 125.9 ± 3.3; Fl1 = 182.3 ± 5.4; Fl2 = 164.8 ± 5.9; Fl3 = 139.6 ± 3.6; Fl4 = 135.7 ± 2.9; Fl5 = 122.2 ± 3; Fl6 = 115.7 ± 2.8; Fl7 = 96.6 ± 2.1; Fl8 = 100.4 ± 2.3; Fl9 = 92.1 ± 3; Fl10 = 88.1 ± 3.9; Fl11 = 83.9 ± 4; Fl12 = 143.3 ± 8.8. (**B**) Ventral view of the head capsule with antennae. (**C**) Close-up view of the apical part of the flagellum (A10–A14). (**D**) Detail of the apical antennomere (A14) characterized by a transverse furrow (black arrowheads) positioned in the medial region of the antennomere. White arrowheads indicate the position of sensilla chaetica. Bar scale: (**A**,**B**) 500 µm; (**C**), 100 µm; (**D**), 20 µm.

*Dryocosmus kuriphilus* females had typical geniculate filiform antennae located on the frontal part of the head capsule, between the compound eyes (Figure 1B). Each antenna was composed of 14 antennomeres, which make a total length of about 1680 µm for the whole antenna (Figure 1A). The antenna was attached to the head capsule through a short (90 µm) scape, which presented an indentation in its basal part. The pedicel was the second antennomere, it was cylindrically shaped and relatively short (about 125 µm). The flagellum, which represented most of the antennal length (about 1450 µm), was composed of the remaining 12 antennomeres. The most proximal antennomeres (particularly A3–A6) were longer when compared with the rest of the antenna (A7–A14) (Figure 1A,C). However, the distal part of the antenna was not differentiated into a well-defined club, as it was often observed for other Hymenoptera families. The apical antennomere (A14) was pointed at the tip and characterized by a transverse furrow in its medial line (Figure 1D).

Six morphologically distinct types of sensilla were observed externally—sensilla placoidea, sensilla chaetica, sensilla coeloconica type-I, sensilla coeloconica type-II, sensilla trichoidea and campaniform sensilla. The main morphological features of antennal sensilla are reported in Table 1.

### 3.1. Sensilla Placoidea (SP)

**Figure 2:**
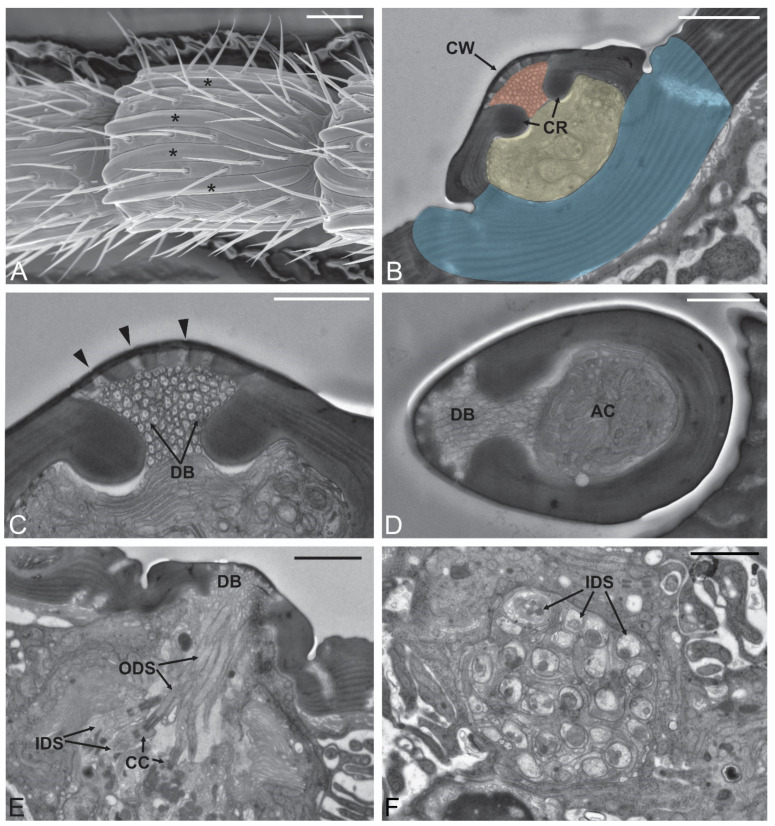
Sensilla placoidea. (**A**) SEM micrograph showing A9 with the presence of numerous sensilla placoidea (*): they can be as long as the antennomere itself. (**B**–**E**) Transmission electron microscopy (TEM) micrographs showing internal features of sensilla placoidea though sections taken at different levels. In (**B**), the sensillum is proximal to its distal end. Different regions were colored to better highlight the different areas. In red is the area occupied by the dendritic branches projected by the outer dendritic segments innervating the sensillum. They completely fill the lumen that is externally outlined by the sensillum cuticular wall (CW), while internally there are two cuticular ridges (CR). Just below these two elements, there is a second area (colored in yellow) that is occupied by the sensillum accessory cells, and at this level is separated from the antennal lumen by a thick cuticular wall (colored in blue). More distally (**D**) the sensillum is separated from the antennal wall but still maintains the separation into two regions. In (**C**), the cuticular pores (black arrowheads) that open on the sensillum wall are presented. (**E**) TEM proximal cross-section showing the sensory neurons innervating the sensillum placoideum. Each sensory neuron is clearly divided into a proximal inner dendritic segment (IDS) and an outer dendritic segment (ODS). Between them, typical ciliary constrictions (CC) are visible. ODS enter the sensillum lumen where they organize in numerous dendritic branches (DB). Each sensillum placoideum is innervated by about 25 sensory neurons, as shown in (**F**). Bar scale: (**A**), 20 µm; (**B**,**E**,**F**), 2 µm; (**C**,**D**), 1 µm.

Sensilla placoidea (SP) were elongated, flattened sensilla, evenly distributed through the flagellum. On the scape and pedicel, SP were absent. A close-up view of the medial region of SP revealed how this area of the sensillum is slightly elevated compared to the lateral regions (Figure 2A). Sensilla placoidea were longitudinally positioned over the antennomeres and were evenly distributed on both dorsal and ventral sides. The number of SP was about 4 on both dorsal and ventral sides making 8 in total.

The length of the SP varied between 70 and 100 μm, with the longest being observed on the proximal antennomeres and on the apical one (A14) (Table 1). Transmission electron microscopy revealed the internal organization of the SP, with the main structural feature represented by the presence of a multiporous cuticular wall (Figure 2C). Cross-sections of SP taken at different points revealed the presence of two longitudinal cuticular ridges which clearly divided the sensilla into two parts. In the outermost, numerous dendritic branches were present, completely filling the lumen and reaching the multiporous sensory area. The innermost space, positioned just below the previous region, is occupied by the accessory cells that projected into this area (Figure 2B–D). Distally, SP were completely separated from the surrounding antennal wall, while they were found merging and being incorporated by the antennal wall itself more proximally. Proximally, SP were internally completely open, allowing the innervating sensory neurons to penetrate inside the sensillum lumen (Figure 2E). Cross-sections taken at the level of the inner dendritic segment showed up to 25 sensory neurons innervating each SP (Figure 2F).

### 3.2. Sensilla Trichoidea (ST)

**Figure 3:**
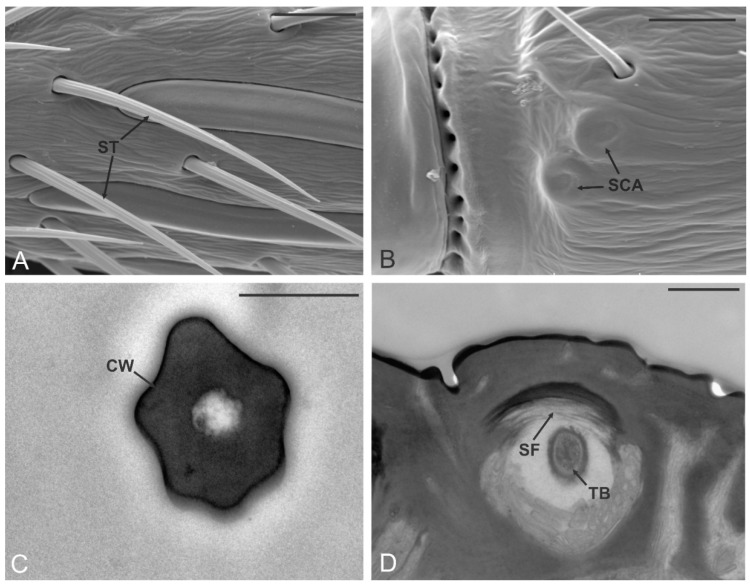
Sensilla trichoidea (ST) and campaniform sensilla. (**A**,**B**) SEM details of the pedicel (A2) and A4 respectively, where it is possible to observe two sensilla trichoidea (ST) and two sensilla campaniformia (SCA). In (**C**) a TEM cross-section through the cuticular peg of an ST: the thick poreless cuticular wall (CW) delimits an internal lumen without sensory neurons inside. In (**D**) a TEM section through the socket of ST: suspension fibers (SF) and a single sensory neuron giving rise to a tubular body (TB) are depicted. Bar scale: (**A**,**B**), 10 µm; (**C**), 0.5 µm; (**D**), 1 µm.

ST were the most abundant sensilla on the antennae. They were present on all antennomeres and distributed on both dorsal and ventral sides. Generally, ST are arranged in lines following the longitudinal axis of the antenna and positioned in the areas of the antennal wall devoid of sensilla placoidea. The number of ST varied between antennomeres. Normally, on the scape and pedicel ST were present in the lower number, while from the third antennomere an increase in their number was recorded. ST were about 30 µm long, with a bristle-like shape. The sensillum tip was pointed which allowed easy identification of ST from sensilla chaetica which were characterized by a blunt tip instead (Figure 3A). The cuticular wall of ST was crossed by longitudinal, shallow furrows for all of its length. TEM data obtained from cross and longitudinal sections taken at different positions showed the presence of a thick cuticular wall with no sensory neurons entering the sensillum shaft (Figure 3C). However, the presence of a single sensory neuron ending with a tubular body and connected with ST base was recorded (Figure 3D).

### 3.3. Sensilla Chaetica (SCH)

**Figure 4:**
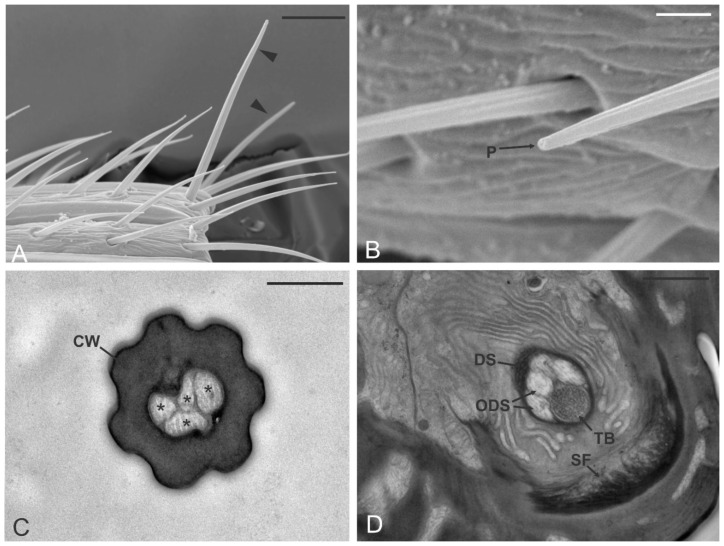
Sensilla chaetica (SCH). (**A**,**B**) SEM pictures showing the sensilla chaetica (arrowheads in (**A**)) typically positioned at the distal margin of one of the antennomeres (in this case A10) and emerging from the rest of the antennal sensilla. In (**B**) a sensillum chaeticum with the apical pore (P) is highlighted. (**C**,**D**) TEM sections of the sensillum revealed the presence of a thick, aporous cuticular wall (CW) and a lumen occupied by four unbranched outer dendritic segments (*). Proximally, at the level of the sensillum socket, sensilla chaetica present suspension fibers (SF) around the socket. The fascicle of sensory neurons is enclosed by a single dendrite sheath (DS) and is made up of the four abovementioned ODS plus a fifth sensory neuron that develops in a tubular body (TB). Bar scale: (**A**), 10 µm; (**B**), 2 µm; (**C**), 0.5 µm; (**D**), 1 µm.

Sensilla chaetica (SCH) were characterized by their peculiar shape and position. Generally, they were observed from the 7th antennomere, mostly positioned on the distal part of the single antennomere. On the apical antennomeres, SCH were distributed in two groups. The first extended medially through the antennomere in coincidence with transverse furrow, while the second was located at the tip of the antennomere (Figure 1D). SCH were easily distinguishable from other sensillar types because of their insertion angle with the antennal wall (around 45°) that made them protrude from the antenna profile (Figure 4A). The number of SCH was 1–3 per antennomere, except for the apical one, where SCH were found in number ranging from 6 to 7. The external cuticle of SCH showed evident longitudinal grooves that run from the base up to the blunt tip. At this point, a single apical pore was observed (Figure 4B). The total length of the sensilla was about 30 µm, while the base diameter was 2 µm. TEM investigations revealed the presence of a thick, poreless cuticular shaft, bordered by evident ridges as a result of the external furrows (Figure 4C). The sensillum lumen housed four unbranched outer dendritic segments. Sections taken at the level of the socket showed the presence of a fifth neuron that ended in the tubular body. The bundle was enclosed by a thick dendrite sheath. At this level, the basal socket of SCH differentiated a joint membrane and suspension fibers (Figure 4D).

### 3.4. Sensilla Coeloconica Type-I (SCO-I)

**Figure 5:**
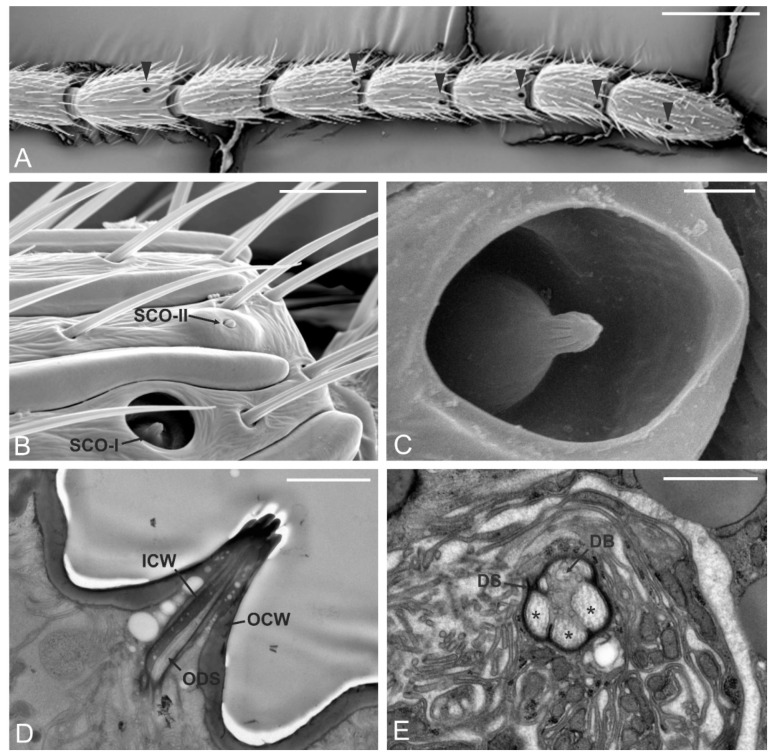
Sensilla coeloconica (SCO). (**A**) SEM micrographs showing the antennomere interval A8-A14. Each antennomere (except A9) presents a sensillum coeloconicum type I (SCO-I) positioned distally on the antennomere (black arrowheads). (**B**) Detail of the distal region of A8, SCO-I can be easily distinguished from the sensillum coeloconicum type II (SCO-II), positioned more distally. (**C**) Close-up view of SCO-I: a grooved peg resides inside the pit that opens externally through a large aperture. (**D**,**E**) TEM micrographs of SCO-I. In (**D**) a longitudinal section through the peg shows the presence of a double wall organization, with an internal cuticular wall (ICW) and an external cuticular wall (OCW). The internal lumen is filled with outer dendritic segments (ODS) of the sensory neurons. In (**E**) a cross-section taken below the cuticular peg: three ODS (*) and a fourth organized into dendritic branches (DB) are visible, all of them enclosed in a single dendritic sheath (DS). Bar scale: (**A**), 100 µm; (**B**), 10 µm; (**C**,**D**), 2 µm; (**E**), 1 µm.

SCO-I showed the typical “peg-in pit” arrangement, with the sensillum (peg) located inside a rounded opening (pit). SCO-I were located on the distal part of each antennomere in the interval A8–A14. Typically, only one sensillum per segment was observed. SCO-I were never observed on the A9 in all the analyzed specimens (Figure 5A). SCO-I external morphology comprised a relatively large cavity, which was circular to oval in shape (6 µm diameter). The peg resided completely within the cavity (Figure 5B,C). This peg presented a conical shape, with a large smooth proximal region and a slender distal part that presented several fingerlike ridges along its length, up to the tip. The ultrastructural investigation of the sensilla revealed double-walled organization, with an external thin cuticular wall and an internal wall housing the outer dendritic segments (Figure 5D). TEM section taken below the peg socket level revealed the presence of three sensory neurons enclosed by thick dendrite sheet. A fourth sensory neuron, for which some dendritic branches were found, was also enclosed in the same dendrite sheath (Figure 5E).

### 3.5. Sensilla Coeloconica Type-II (SCO-II)

SCO-II were generally located on the distal part of the 6th, 8th, and 10th antennomere. Occasionally SCO-II could be observed next to the SCO-I. SCO-II were organized as rounded, flat, or slightly elevated cuticular plates, with a peg protruding from the center (Figure 5B). The sensilla were about 5 µm wide, while the length of the pit was about 8 µm. No TEM data were available to describe ultrastructural features of this sensillum.

### 3.6. Sensilla Campaniformia (SCA)

The sensilla campaniformia (SCA) were located at the basal part of the antennae, generally from the pedicel up to the 5th antennomere. SCA were disk-like structures, with an outer ring encircling a flattened, smooth central area (Figure 2B). They showed a diameter of about 5 µm. In a few specimens, SCA were observed up to the 8th antennomere. No TEM data were available to describe ultrastructural features of this sensillum.

### 3.7. Ovipositor

**Figure 6:**
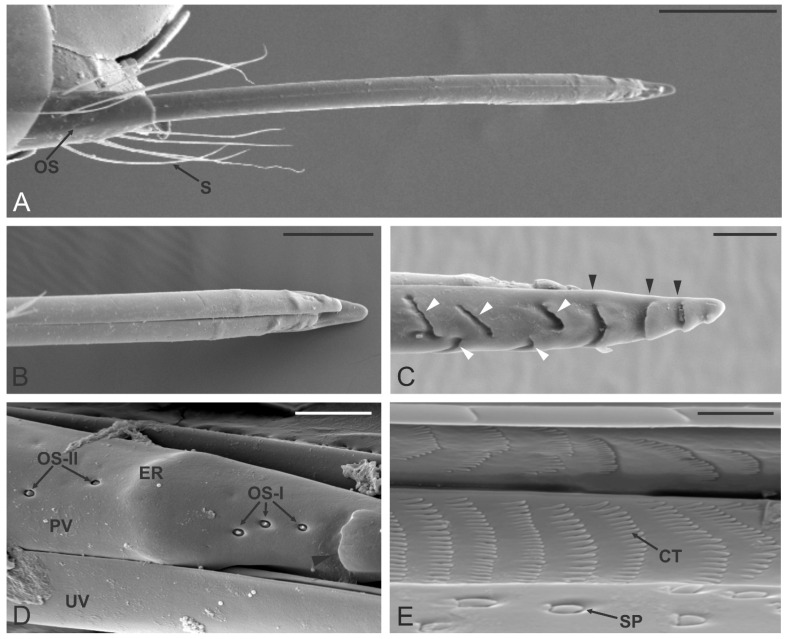
*Dryocosmus kuriphilus* ovipositor. (**A**) General view of the true ovipositor, at the base a tuft of elongated setae (S) and the third pair of valves (the ovipositor sheath OS) are visible. (**B**,**C**) SEM micrographs of the paired valves (in (**B**)) and unpaired valve (in (**C**)). The latter presents transverse furrows as a single (black arrowheads) or double slots (white arrowheads). (**D**) Close-up view of the apical part of one of the paired valves (PV) still attached to the unpaired valve (UV): note the presence of a single furrow (black arrowhead) and the elevated region (ER). The area delimited by the furrow and the elevated region presents three ovipositor sensilla type I (OS-I), while a couple of ovipositor sensilla type II (OS-II) are observed just behind the ER. (**E**) SEM micrograph showing a detail of the internal organization of the paired valve: several ctenidia (CT) arranged in the parallel pattern can be observed, as well as some scale-like pegs (SP) positioned on the sliding surface of the valve. Bar scale: (**A**), 100 µm; (**B**), 50 µm; (**C**), 20 µm; (**D**), 10 µm; (**E**), 5 µm.

**Figure 7:**
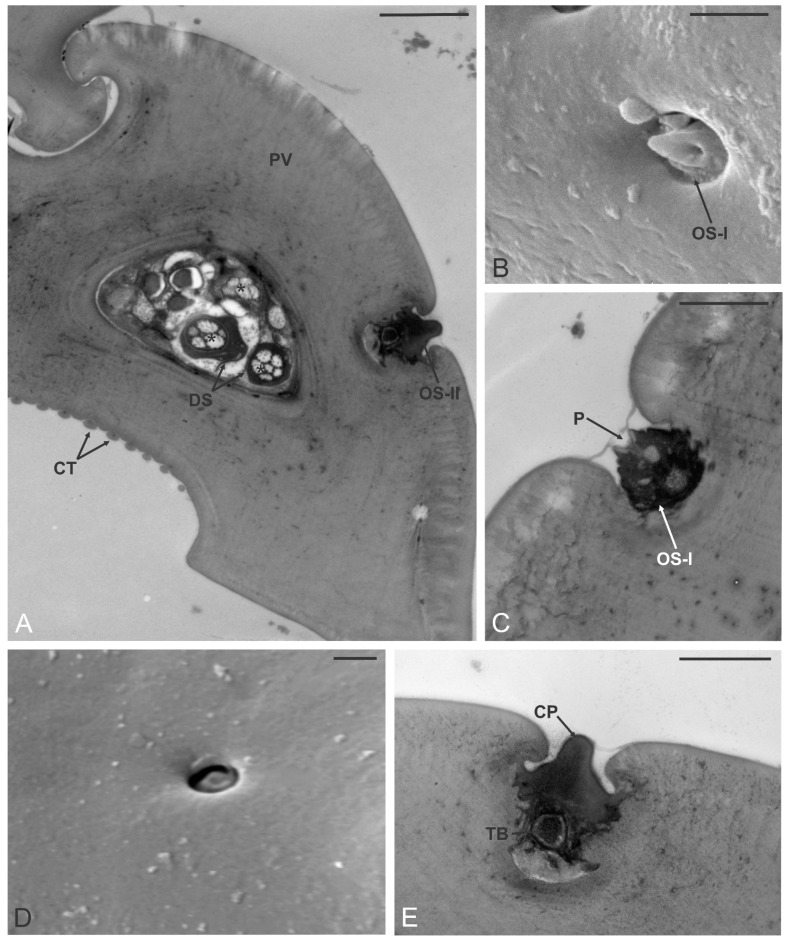
*Dryocosmus kuriphilus* ovipositor. (**A**) TEM cross-section of one of the paired valves (PV): the external side presents a single ovipositor sensillum type II (OS-II), while the internal side is occupied by a single row of ctenidia (CT). The PV lumen reveals the presence of three bundles of sensory neurons (*), each one comprised of 5–6 sensory neurons enclosed in a thick dendrite sheath (DS). (**B**) SEM close-up view of an OS-I: a well evident apical pore is observed. (**C**) TEM longitudinal section of OS-I: the apical pore (P) is revealed. (**D**) SEM picture of an OS-II. (**E**) TEM longitudinal section of an OS-II: the peg (CP) is made of solid cuticle without pores and reveals the presence of a single sensory neuron ending in a tubular body (TB). Bar scale: (**A**), 2 µm; (**B**–**E**), 1 µm.

The ovipositor of *D. kuriphilus* followed the basic structural organization found among other Hymenoptera Terebrantia. In common with those of all other Hymenoptera, this sophisticated structure comprises a pair of valves (located at the base of the abdomen) that provide protection to the proper ovipositor. The true ovipositor is made of a single upper part (unpaired valve) and two lower paired valves (Figure 6A–C). The whole structure appeared long and slender (about 550 µm long), with a constant diameter (about 25 µm) for its entire length. The very apical part of the three valves was narrow and ended in a fine tip which was rounded in the unpaired valve, while it appeared sharper in the paired valves. The unpaired valve showed externally the presence of transverse furrows which appeared as a single slot (the first three counted from the tip) or as double separated slots (Figure 6C). This cuticular organization gave to the valve a saw-like appearance. The paired valves were pointed, with a single transverse slot located near the tip, and a swollen region more proximally; these two structures delimited a smooth area where three small pegs were found (Figure 6D). Close-up view of these pegs revealed a “peg-in-pit” organization, with the presence of an irregular elliptical apex with a pointed margin oriented towards the ovipositor tip. A single apical pore was observed (Figure 7B). We defined these sensilla as ovipositor sensilla type I (OS-I). TEM sections taken below the third OS-I revealed the presence of three groups of sensory neurons, each one surrounded by thick dendrite sheath. Each group, that belonged to the three OS-I, presented 5–6 sensory neurons (Figure 7A). Longitudinal section of OS-I showed the presence of a conical peg with a single apical opening (Figure 7C). Looking at the paired valve, just below the elevated area a second type of sensory structure was found, but with a different external shape. These sensilla, named as ovipositor sensilla type II (OS-II), appeared like small, flattened pegs that resided in a small opening in a way that they were located just below the cuticle outline (Figure 7D). TEM sections revealed the presence of a thick cuticular peg with a single sensory neuron at the base, ending in a tubular body (Figure 7E). The main morphological features of the ovipositor sensilla are reported in the Table 1.

Internal walls of the three valves that together made the egg canal showed the presence of two different type of structures. The first type of structures was regularly distributed over the egg canal in spaced lines (Figure 6E). They had the shape of a flattened peg, with the free tip, oriented toward the ovipositor tip. The second type of structures were located at the edge of each valve, where the interlocking with another valve occurred. These structures were flattened scale-like pegs arranged in a line, in shallow depressions that housed the peg completely (Figure 6E).

## 4. Discussion

The Cynipidae, also known as gall-wasps, represent a rich and diverse family of Hymenoptera, comprising about 1400 described species [31]. Despite their importance there are a few studies related to the ultrastructure organization of their sensory systems [25,32,33,34]. In this paper, we reported fine structural data of the antennae and ovipositor sensilla in female individuals of the investigated species: due to *D. kuriphilus* reproduction through thelytokous parthenogenesis, the population is entirely composed of females.

The antennae of *D. kuriphilus* are of the filiform type, and thus follow the general composition found in other cynipids [25]. A similar antennal organization was reported within the Cynipoidea also in the family Figitidae as well as in the Cynipidae family [25], being a common feature within the group. Within the Hymenoptera Terebrantia (parasitic wasps) a different female antennal organization was often reported (i.e., families. Scelionidae, Trichogrammatidae), with a distinct club-like structure leading to an obvious sexual dimorphism (with males having filiform antennae) [35,36,37].

In *D. kuriphilus*, we reported similar types and distribution of sensilla as in other investigated Cynipoidea species [25,34,36,38]. Specifically, sensilla placoidea were present from the third antennomere. In several species of Figitidae, absence of sensilla placoidea on the third antennomere was reported [25]. However, this is not consistent throughout the family [25,34,36]. The shape of the SP (elongated, plate-like, multiporous sensilla) is similar to that of SP described in other Cynipoidea species, in both males and females. Sensilla placoidea were reported also in Hymenoptera Aculeata, but in this suborder they appear as small, oval areas instead of long, elongated plates [25,28,38]. For SP, olfactory function was proposed [28]. The number of sensory neurons innervating each SP varies among Hymenoptera species. In *Aphidius smithi* Sharma and Subba Rao (Aphidiidae) it is 37 [39], in *Itoplectis conquisitor* (Say) (Ichneumonidae) it is 27 [40], whereas in *Apis mellifera* L. it varies from 12 to 18 [41]. In *D. kuriphilus*, we observed a total of 25 neuronal cells per SP. Such variability is likely related with the peculiar habits of each species. For *D. kuriphilus*, we can hypothesize that SP are involved in the host-searching behavior, through perception of specific volatile organic compounds (VOCs). Among VOCs, green leaf volatiles (GLVs) represent one important group of plant volatiles which are induced by herbivory attack, fungal or bacterial infection, as well as by abiotic stress or mechanical damage [42]. Green leaf volatiles are perceived by insects from a long distance as a result of their higher volatility [43].

Behavioral investigations on *D. kuriphilus* showed no attraction towards undamaged seedlings, intact twigs, or freshly mechanically damaged tissues, on the contrary, attraction was observed towards twigs with old mechanical damage [20]. Such differential response was likely the results of a change in the GLVs composition between undamaged and damaged host plants, as chemical analysis revealed significant qualitative and quantitative variations in the GLVs emission [20]. Specifically, the volatile blend collected one hour after mechanical damage shows reduction of (Z)-3-hexenyl acetate and increase in the hexanol, and (E)-2-hexanol [20]. Based on their structural morphology, distribution and abundance we hypothesize that sensilla placoidea could play a prominent role in the perception of GLVs.

Together with the SP, sensilla trichoidea represent the most abundant type of sensilla on *D. kuriphilus* females. Among Hymenopteran species, ST show high morphological variation [32,33,44,45]. Polidori and Nieves-Aldrey [25] reported five distinct types of ST in Cynipoidea species. In the case of *D. kuriphilus* we have observed only one distinct type of ST. Sensilla trichoidea in [25] were denoted as ST-C. Moreover, the mean length of ST reported in the same study was smaller when compared to the data presented here. This could be an indication of an intraspecific variation occurring in this species as regards sensilla size. However, these data should be correlated to the body size of measured in-dividuals as well, to rule out the possible effect of body size itself.

Generally, ST are associated with mechanoreceptive or olfactory functions, based on their morphological features. In the case of *D. kuriphilus* we exclude possible olfactory function because of the presence of the tubular body and lack of pores on its cuticular wall (characteristic exclusively found in olfactory sensilla). Because of this, we propose for ST a role as mechanoreceptors, involved in the location of a suitable oviposition site. In the cynipid *Diplolepis rosae* L., the female displays characteristic antennation [21], lasting 5–60 min. During this process, the female antennates the substrate to locate an expanding bud, after which she positions herself for oviposition. In *D. kuriphilus* a similar behaviour was reported [22], thus, we hypothesize an active involvement of the antennae in locating an oviposition site. In this context, ST are involved in detecting the tip of the expanding bud.

Sensilla chaetica (SCH) were present on both sides of the antennomeres. However, we could not find a consistent pattern regarding the number and position of SC, except that they are generally located in the distal part of each antennomere. In a previous work conducted on *D. kuriphilus* these sensilla were denoted as Sensilla trichoidea type B [25]. These were found to be smaller in length when compared to SCH. Nevertheless, the mean length of ST and SCH in both studies was similar. Moreover, in several cases, SC-a was recognized as long sensilla trichoidea [32], or sensilla basiconica—type 1 [44]. The sensilla chaetica are positioned perpendicularly on the cuticle, thus allowing for easier and direct contact with the substrate. Based on their ultrastructural organization, for SCH a role in chemo- (gustatory) and mechanosensory detection is proposed. Several studies reported different compositions and concentrations of chemical substances between galls and other tissues. Indeed, higher levels of phenolic compounds in the galls induced by *Leptocybe invasa* Fisher and La Salle (Hymenoptera: Eulophidae) compared with control tissues have been reported [7,23]. Moreover, a meta-analysis conducted on secondary metabolites induced by galling insects found a significantly higher level of tannins and phenolic compounds [23]. Flavonoids represent a large group of phenolic compounds, which are known to affect insects feeding and oviposition [46,47]. In the case of monarch butterfly, *Danaus plexippus* L., it was demonstrated that flavonoids have a stimulative effect on the female oviposition. Moreover, electrophysiological investigation showed that mid-tarsal and antennal sensilla responded towards flavonoids [48]. Although there are no data available regarding the antennal response to gustatory stimuli in Cynipidae, we hypothesize that sensilla chaetica can be involved in testing the suitability of the host plant.

Sensilla coeloconica (SCO) are characterized by a peg-in-pit structure. Three different peg-in-pit sensilla have been described previously, sensilla ampullacea, sensilla coelocapitula and sensilla coeloconica. Our studies found two distinct types of SCO on *D. kuriphilus*’s antennae, named SCO-I and SCO-II. In the previous study conducted on Cynipidae these sensilla were termed as SCo-A and SCo-B respectively [25]. The SCO-I were present in higher number than SCO-II and distributed on the ventral side of the distal antennomere. They were frequently accompanied by the SCo-II. In general, one sensillum per antennomere was observed. This is in line with other studies conducted on Cynipoidea and locusts (Orthoptera) [49]. Moreover, in all analyzed specimens we did not observe SCO-I on the 8th antenommere. No microtrichia were observed around these sensilla, as reported in the case of biting midges (Diptera: Ceratopogonidae) [50]. Similar structural organization of the SCO-I was observed in *Atta vollenweideri* Forel, a leaf-cutting ant [51], *Scaphoideus titanus* Ball (Hemiptera: Cicadellidae) [52], and *Locusta migratoria* L. [49]. Sensilla coeloconica-II in *D. kuriphilus* females resemble those found in honeybee, *Apis melifera* L., on which they were denoted as s. coelocapitula [53]. These sensilla are also reported as poreless sensilla (np). The typical peg-in-pit structural organization of the sensilla coeloconica is assumed to be related with sensilla protection from the mechanical damage and/or humidity balance inside the peg, which can affect the absorption of odor molecules [53,54]. Several functions were proposed for these sensilla, i.e., thermoperception, thermo-hygroperception, a combination of thermo-chemo sensitivity or solely chemoperception [51]. Moreover, these sensilla were reported to be involved in the perception of CO2 [55]. For SCO-I we propose a possible olfactory function because of its double-walled organization and the presence of pores, however, a combined chemo-thermosensitive function cannot be ruled out. In the case of SCO-II we hypothesize a thermo-hygroreceptive function. The ultrastructural investigations of thermo-hygroreceptive sensilla have shown significant uniformity between analyzed species. Usually, for SCO-II a triad of sensory neurons in observed, with two unbranched dendrites that extend into the lumen of the peg and a third lamellated dendrite ending at the base of the peg [56]. However, a study conducted by Schneider et al. [54], found a sensillum in which all three dendrites are unbranched.

Lastly, we observed several SCA unevenly distributed over the proximal part of the antenna. They were positioned singularly or in pairs. In general, SCA are associated with the perception of cuticular deformation by external factors or self-generated movement [57]. Thus, they are often located where such deformation frequently occurs, legs, wings, halteres or near the joints [57,58]. Previous investigations reported SCA ability to detect the direction and speed of antennomere movements [59]. Thus, we assume that SCA detect forces produced by antennal movement based on which female is able to identify antennal position. This proprioreceptive function is also paired with so-called Böhm bristles, a series of short mechanosensory pegs usually located in the joint area between the scape and head capsule [59]. Moreover, these sensilla are known to be involved in the perception of vibrations used by insects to communicate with conspecifics [60,61].

Oviposition represents the last step in the reproduction cycle of an insect. It is considered as a crucial step, in which a female needs to locate, assess, probe, and lay an egg and in some cases inject venom in a suitable host [62,63,64]. In Hymenoptera Terebrantia, the general morphology of the ovipositor is mostly conserved among families, and it is composed of a fused upper valve and two ventral pair of valves [58,61]. The exception to this organization can be found among Ophiniformes (Ichneumonidae), in which the valves of the upper pair are almost completely separated, except at the apex [63,65]. Despite this general recurrent organization of the ovipositor, differences can be found among Hymenoptera families in the position and number of serrations present on the different valve, in the level of sclerotization and in the number and variety of sensilla. In most Ichneumonidae and Braconidae, serrations are located on the ventral valves, which is different from our findings, where we found serrations located mainly on the dorsal (unpaired) valve and one level of serrations on the ventral (paired) valves. Similar findings were observed in the case of *Leptopilina heterotoma* Thomson, 1862 (Eucoilidae) [24]; Agaonidae [62,66]; *Xestophanes potentillae* (Retzius, 1783), *Synophrus politus* Hartig, 1843 (Cynipidae) [67]. The proposed function for serrations is mainly related to substrate penetration. It has been proposed that the position of serrations is correlated with the biology of species, and the ovipositing mechanism. Species which oviposit shallow in the substrate, in an exposed host or glue the eggs on the leaf surface show no or a low number of teeth on the ovipositor compared to those which need to drill woody material [62,63,68]. Moreover, metal-enrichment (Zn, Mn, Cu) increases ovipositor sclerotization and eventually successful substrate penetration [67]. The level of sclerotization is proportional to the pressure required to penetrate through the substrate. No reinforcement was found on the ovipositor of *D. kuriphilus* [67]. Similarly, in other gall-inducing species, sclerotization was absent, compared to inquilines, in which different degree of sclerotization was observed [67]. On insects’ ovipositor, several types of sensilla were reported. Distribution and variation of sensilla are related to the oviposition modalities. For example, in those species in which only the ovipositor tip penetrates sensory structures are often grouped towards the apex. In contrast, species in which the ovipositor probes deep in the substrate have sensilla distributed along the structure [64]. On the external surface of *D. kuriphilus* ovipositor we found two types of sensilla. Distally, a group of three OS-I was observed. These sensilla presented a single apical pore, therefore we hypothesize a gustatory function. This is consistent with observations in several species where presence of chemosensory sensilla at the apical part of ovipositor was reported [66,69,70,71]. Below gustatory sensilla, an elevation on ovipositor was observed, after which, ovipositor sensilla type II were observed, for which we hypothesized a mechanoreceptive role. When lower and upper valves are locked through olistheter mechanism, they create a lumen which represents the egg channel. On *D. kuriphilus* egg channel walls we reported the presence of peg-like structures, that can be assimilated to ctenidia, saw-like structures arranged in multiple rows [72]. Ctenidia represent a common structure on internal walls of the egg channel. Number of ctenidia types varies between species, mainly in range from 1–2, however in a few cases up to 4 types were observed [64,71,72,73,74]. It is presumed, that ctenidia play a role in egg movement along the channel and in preventing backward movement. Moreover, a lubricating effect which eventually decreases the friction of eggs or maintain the amount of liquid within the egg canal has been proposed [74]. Besides ctenidia, additional structures on the sliding surface between dorsal and ventral valves were observed. Such structures are likely not involved in the egg movement as they are not in direct contact with it. We hypothesize that they could function as proprioreceptors, receiving information about the relative position of the valves themselves.

The steering and ovipositing mechanism for a koinobiont species was described by Boring et al., [74]. In this work, the mechanism was divided into four phases: (i) ovipositor penetration of the substrate; (ii) locking mechanism; (iii) mechanism of egg movement along egg canal; and (iv) egg-laying and ovipositor withdrawal. We hypothesize that differences in the ovipositor penetration of the substrate could be mainly related to the penetrating mechanism. Boring et al. [74] proposed that after the initial steering of the substrate, which is done by a series of ventral valves movement, the dorsal valve penetrates the substrate up to the notch level. As a result of different sensilla position and ovipositor’s barbs, we hypothesize that in *D. kuriphilus* the depth up to which the ovipositor will penetrate substrate would depend on the ventral valves position (differently from the previous study, in which depth of penetration is hypothesized to be level of the notch level on the dorsal valve). We support our idea because of the mechanoreceptive sensilla position on ventral valves. Namely, when penetrating the substrate, the gustatory sensilla of ventral valves will assess the suitability of the tissue. If the ovipositor goes deeper, the mechanoreceptive sensilla (located more proximally and behind the valve notch) will trigger backward movement. This hypothesis is also supported by *D. kuriphilus* biology to oviposit eggs exclusively in the apical portion of the bud, between internal young leaf primordials [21,22].

## 5. Conclusions

In this paper, we report the fine structural organization of antennal and ovipositor sensilla in *D. kuriphilus*. Based on the ultrastructural data, we confirm the ability of *D. kuriphilus* to perceive both olfactory and gustatory cues thanks to the presence of sensilla placoidea (with an olfactory function) and sensilla chaetica (with a double mechano-gustatory function). Moreover, the ovipositor also presents an array of sensilla (of the gustatory and mechanosensory type) allowing precise discrimination of the host plant and the oviposition depth.

## Figures and Tables

**Table 1:** Morphological features of the antennal and ovipositor sensilla in *Dryocosmus kuriphilus* female (mean ± SE).

Sensilla Features	Sensilla Type							
	Antenna						Ovipositor	
	Trichoidea	Chaetica	Coeloconica-I	Coeloconica-II	Placoidea	Campaniformia	SO-I	SO-II
Length (µm)	31 ± 1.4	28 ± 1.2	-	-	81 ± 3.6	-	-	-
Diameter (µm)	-	-	6.2 ± 0.3	5.3 ± 0.7	-	4.1 ± 0.3	1.2 ± 0.1	1.3 ± 0.1
Pores	Aporous	Uniporous	Uniporous	Aporous	Multiporous	Aporous	Uniporous	Aporous
Wall	Single thick	Single thick	Double thick	-	Single thin	Single thick	Single thick	Single thick
Neurons (n)	1	5	4	-	25–30	1	5–6	1
Cuticle	Grooved	Grooved	Grooved	Smooth	Smooth	Smooth	Smooth	Smooth
Tip	Pointed	Blunt	Blunt	Blunt	-	-	Blunt	Blunt
Base	Socketed	Socketed	Unsocketed	Unsocketed	Unsocketed	Unsocketed	Unsocketed	Socketed

## Data Availability

All microscopy data are available from the authors upon request.
